# Cobalt-modified graphitic carbon nitride nanosheets for the photocatalytic degradation of diclofenac

**DOI:** 10.3389/fchem.2026.1901405

**Published:** 2026-07-27

**Authors:** Junyan Pan, Luyao Sun, Ekaterina Kozlova, Binhong Qu, Yang Qu

**Affiliations:** 1 Key Laboratory of Functional Inorganic Materials Chemistry (Ministry of Education), School of Chemistry and Materials Science, International Joint Research Center and Lab for Catalytic Technology, Heilongjiang University, Harbin, China; 2 Federal Research Center, Boreskov Institute of Catalysis, Novosibirsk, Russia

**Keywords:** charge separation, cobalt species, diclofenac, g-C_3_N_4_ nanosheet, visible-light degradation

## Abstract

Diclofenac (DCF), as a challenging new type of pollutant, is resistant to removal by photocatalysis due to the poor visible-light utilization and sluggish charge separation. In this study, cobalt (Co) modified graphitic carbon nitride (3Co/CCN) was successfully synthesized using a cyanuric acid (CA)-induced dispersion of cobalt phthalocyanine (CoPc) within urea through mechanochemical method and the subsequent thermal polymerization route. Under visible-light irradiation, the 3Co/CCN displayed 2.8 times higher DCF photodegradation than that of CCN, accompanied by a 21.4-fold enhancement in reaction kinetics rate. It also demonstrated excellent photocatalytic performance in complex environmental conditions with notable photocatalytic stability. The incorporation of Co species (Co^2+^/Co^3+^) was found to improve the separation of electron-hole pairs, as confirmed by photoluminescence spectroscopy and photoelectrochemical characterization. This study introduces an efficient and cost-effective strategy for the effective removal of DCF from wastewater.

## 1 Introduction

Pharmaceuticals and personal care products (PPCPs), represent a novel category of environmental pollutants that pose significant challenges for global water pollution control due to their ubiquitous presence in aquatic environments and the potential ecological risks they entail (Richardson and Ternes, 2018). Diclofenac (DCF), a non-steroidal anti-inflammatory drug (NSAID) among the most frequently prescribed pharmaceuticals globally, is widely recognized as a representative PPCPs emerging contaminant (Carena et al., 2025). DCF is notoriously difficult to remove effectively through traditional wastewater treatment processes, leading to ongoing contamination of groundwater and drinking water sources (Nosek and Zhao, 2024). The European Union has designated DCF as the first NSAID to be included on its priority monitoring list (Verlicchi et al., 2012). Therefore, it is critical to develop efficient technique to degrade DCF in water environment.

Photocatalysis technology that effectively harnesses abundant, clean, and sustainable solar energy for conversion into chemical energy, has garnered considerable research interest in recent years, particularly for its potential applications in environmental pollution remediation (Cao et al., 2025; Hu and Zhu, 2024). Among various photocatalysts, graphitic carbon nitride (g-C_3_N_4_), a metal-free semiconductor that is responsive to visible-light, has emerged as a particularly promising candidate due to its easy structural tunability, excellent chemical stability, and inherent environmental benignity (Yang et al., 2022). However, suboptimal charge separation capability and a restricted number of reactive sites in g-C_3_N_4_ when combined with DCF constrain its applicability in the degradation of DCF (Liu et al., 2019). Research has demonstrated that the integration of dispersed metallic cobalt (Co) significantly enhanced the photogenerated trapping activity, thereby substantially reducing the recombination of electron-hole pairs of the g-C_3_N_4_ matrix (Li et al., 2023; Tang et al., 2024). Moreover, the incorporation of Co modified the original planar conjugated structure of g-C_3_N_4_, inducing lattice distortion and reducing its long-range order. The 3d orbitals of Co hybridized with the 2p orbitals of nitrogen (N) atoms in g-C_3_N_4_, resulting in the formation of new intermediate electronic energy levels within the intrinsic band gap of g-C_3_N_4_ (Liu X. M. et al., 2025; Yang et al., 2020). Consequently, this reduced the minimum photon energy, leading to an extension of visible-light absorption and an enhancement in the capability for photoelectron transfer (Zhang X. et al., 2026).

The fundamental structural unit of g-C_3_N_4_ is a tris-s-triazine ring, wherein each unit comprises rich N atoms possessing lone pairs of electrons. This configuration offers a more favorable coordination environment for the immobilization of Co active sites (Qin et al., 2023). The conventional approach of impregnating metals onto pre-synthesized g-C_3_N_4_ often leads to the formation of unstable metal sites that are susceptible to detachment, thereby compromising the catalyst’s stability. In contrast, employing a pre-assembled supramolecular precursor strategy, which initially incorporates metal ions within the supermolecular precursor network, followed by thermosetting polymerization, can effectively anchor the metal ions and prevent metal atom aggregation (Qu et al., 2023). Cobalt phthalocyanine (CoPc) is a planar, conjugated macrocyclic molecule characterized by a planar Co–N_4_ coordination structure, making it an ideal Co source (Liu et al., 2026). Moreover, in our previous research, we found that hydroxyl groups could enhance the dispersion of CoPc (Sun et al., 2020). Consequently, the selection of CoPc as the Co source, coupled with the simultaneous incorporation of functional molecules containing hydroxyl groups, can enhance the effective dispersion of CoPc within the precursors used for the synthesis of g-C_3_N_4_. The polymerization of this precursor, characterized by uniformly dispersed Co, is anticipated to yield a stable Co site-modified g-C_3_N_4_.

In this study, we have successfully synthesized Co-modified g-C_3_N_4_ (3Co/CCN) by initially dispersing CoPc within urea through a mechanochemical method induced by cyanuric acid (CA), followed by a thermal polymerization process. This synthesis resulted in Co active sites being uniformly distributed on the g-C_3_N_4_ matrix, accompanied by a large specific surface area and a thinner layered structure (∼3.6 nm). As expected, under LED white light illumination, the photocatalytic degradation activity of 3Co/CCN for DCF exhibited an enhancement of 2.8 times relative to pristine CCN, while the reaction kinetics demonstrated a 21.4-fold increase. Moreover, characterization analyses indicated that the incorporation of Co results in Co^2+^/Co^3+^ states, which expanded visible-light absorption range and improved charge separation and transfer processes within CCN, thereby significantly augmenting its photocatalytic activity. This work presents a viable approach for the metal modification of g-C_3_N_4_, thereby enhancing the practical applicability of g-C_3_N_4_ materials in the photocatalytic degradation of emerging pollutants.

## 2 Experiment section

### 2.1 Synthesis of Co/CCN, CCN and CN

Co/CCN was synthesized via a one-pot thermal condensation strategy followed by controlled acid etching. Add 7.5 g of CA and a certain amount of CoPc to a clean agate mortar, and thoroughly grind at room temperature. Then add 7.5 g of urea and grind them evenly to achieve complete mixing. After grinding, the sample was transferred to a porcelain boat, flattened and compacted. It was then placed in a muffle furnace and heated at a rate of 2.3 °C per minute to 550 °C, and keeping at this temperature for 4 h. After the thermal polymerization reaction completed, stop heating and allow the muffle furnace to cool down naturally to room temperature. A certain mass of the thermally polymerized product was weighed and dispersed in a 5 mol·L^−1^ HNO_3_ solution of a certain volume so as to remove the unreacted precursors and oligomers intermediates. After ultrasonic treatment for 30 min, it was heated at 70 °C for 2 h, then cooled at room temperature and stirred for washing with deionized water until neutrality was achieved. Afterwards, it was washed with anhydrous ethanol and dried in a vacuum oven to obtain the sample. The samples with Co additions at mass percentages of 1%, 3%, 5% and 7% were designated as 1Co/CCN, 3Co/CCN, 5Co/CCN and 7Co/CCN, respectively.

For comparison, the samples prepared using urea and CA without the incorporation of CoPc via the aforementioned method are named CCN. The samples prepared using urea as the raw material through the aforementioned method are named CN.

### 2.2 Characterization

The morphology and structure of the samples were characterized using scanning electron microscopy (SEM, Hitachi, S-4800, JPN) and transmission electron microscopy (TEM, JEOL, JEM-2010, JPN). The surface morphology and thickness of the samples were characterized using atomic force microscopy (AFM, Bruker, Multimode 8, DE). The crystal structure and phase composition of the as-prepared catalysts were characterized via X-ray powder diffraction (XRD, Bruker, D8 Advanced, DE). The measurements were performed using Cu Kα radiation (λ = 0.15406 nm) at a tube voltage of 40 kV and tube current of 50 mA. A continuous scanning mode was employed, with a 2θ scanning range of 5°∼80° and a scanning rate of 6°min^−1^. The Fourier-transform infrared (FT-IR, Bruker, Equinox 55, DE) spectra were collected with a Thermo Scientific Nicolet iS50 FT-IR spectrometer, KBr as the diluents. The X-ray photoelectron spectrometer (XPS, VG, ESCALAB, UK) was used with magnesium Kα as the excitation source (300 W). The binding energy of the surface adventitious carbon C1s (284.6 eV) was used for calibration. Nitrogen adsorption-desorption isotherms were taken on a Quanta chrome-automated gas sorption analyzer (BET, Quantachrome, Autosorb-iQ-C-AG-TCD, USA). Photoluminescence (PL) spectra were carried out on a fluorescence spectrophotometer, LS55 Perkin-Elmer, with an excitation wavelength of 332 nm. The solid/liquid ultraviolet-visible (UV-Vis) absorption spectra of the samples were measured using UV-2700 spectrophotometer, Shimadzu Corporation, JPN. The surface charge properties of the sample particles were tested using a particle size analyzer (Zeta, Malvern Panalytical, Zetasizer Nano ZS90, UK). The photoelectrochemical properties of the samples were characterized by Electrochemical reduction measurement (Ivium Technologies B.V., V13806, NLD).

## 3 Results and discussion

### 3.1 Structural characterization

**FIGURE 1:**
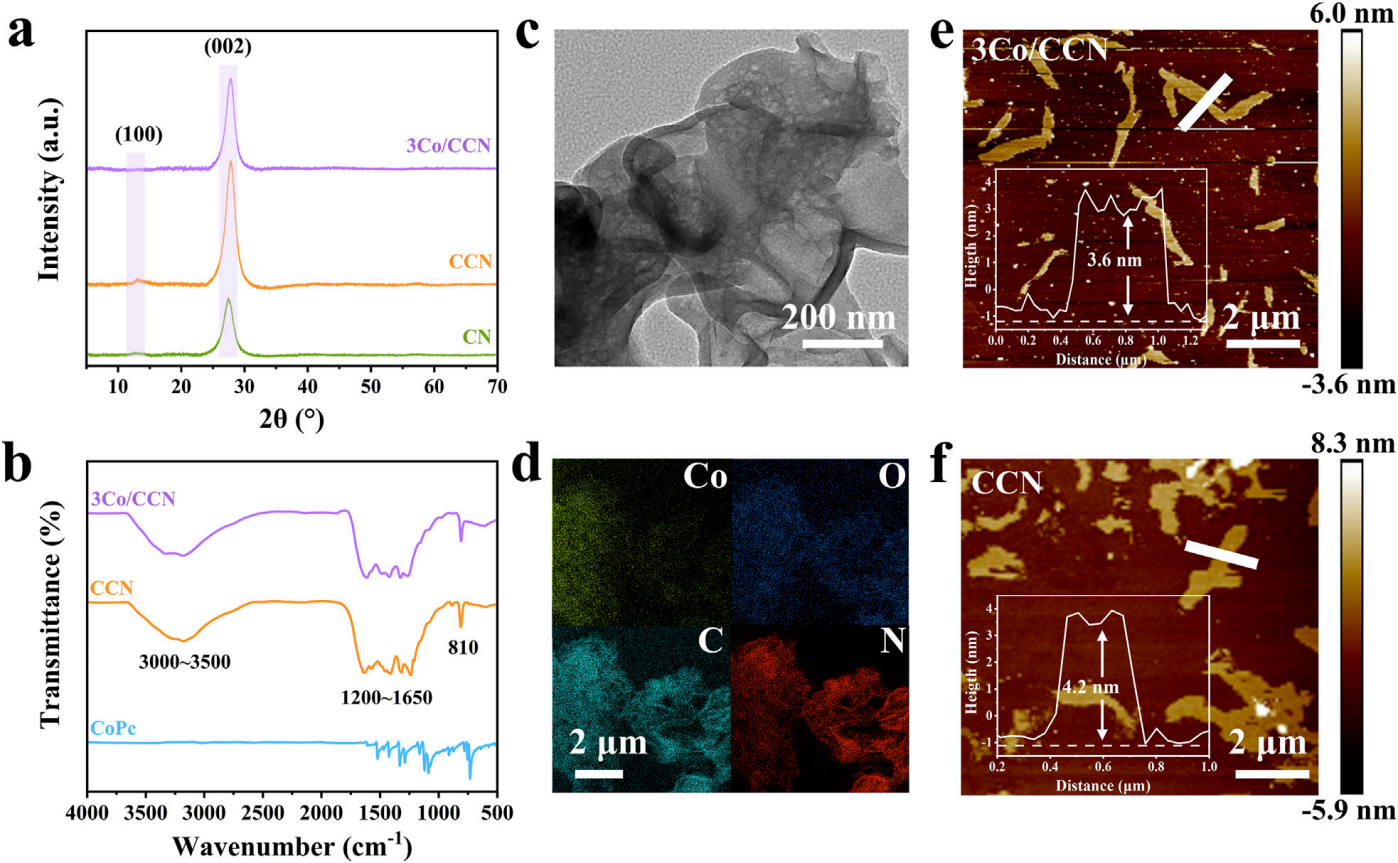
**(a)** XRD patterns of 3Co/CCN, CCN and CN. **(b)** FT-IR spectrum of 3Co/CCN, CCN and CoPc. **(c)** TEM image and **(d)** EDS elemental mapping of 3Co/CCN. AFM images of **(e)** 3Co/CCN and **(f)** CCN, with corresponding height profiles shown in the insets.

In this study, CA was employed as a hydrogen-bonding mediator to facilitate the dispersion of CoPc within urea. This process was substantiated by the UV-Vis absorption spectra (Supplementary Figure S1), which demonstrated that the integration of CA via mechanical-milling with CoPc resulted in a 12 nm red shift and broadening of the Q-band absorption peak, in comparison to CoPc alone. These alterations provided evidence that CA molecules facilitate molecular-level dispersion of CoPc during mechanical grinding, thereby enhancing the planar conjugation of CoPc (Chu et al., 2020). The Co/CCN material was subsequently synthesized by calcining the obtained Co-CA-urea supramolecular precursor followed by acidification. In contrast, control samples without Co modification (CCN) and those lacking both CA and Co modification (CN) were also prepared. Figure 1a presents the XRD patterns of these samples. The data indicate that both CCN and CN exhibited two characteristic diffraction peaks of g-C_3_N_4_ at approximately 13.0° and 27.5°. These peaks correspond to the (100) plane, which is associated with the in-plane ordering of tri-s-triazine units, and the (002) plane, which arises from the interlayer π–π stacking of conjugated aromatic frameworks, as referenced by JCPDS No. 87-1526. This result indicates that the fundamental crystal structure of g-C_3_N_4_ remains unchanged during the synthesis of CCN. Notably, the intensity of the diffraction peak corresponding to the (100) crystal plane in 3Co/CCN has significantly decreased. This reduction is attributed to the incorporation of Co, which has resulted in the disruption of the layered structure and the triazine ring structure present in g-C_3_N_4_ (Cheng et al., 2025). As shown in Figure 1b, FT-IR spectra display a broad absorption band in the 3000∼3500 cm^−1^ range, which is attributed to the N–H stretching vibrations of terminal–NH_2_ and bridging–NH–groups. Characteristic peaks occurring between 1200∼1650 cm^−1^ are ascribed to C=N–C stretching modes within the tri-s-triazine/heptazine framework. Additionally, a sharp peak at 810 cm^−1^ corresponds to the out-of-plane bending vibration (as opposed to stretching) of heptazine units. Notably, no diagnostic absorption bands associated with the phthalocyanine macrocycle, are evident in the spectrum of 3Co/CCN, indicating the complete thermal decomposition of CoPc during calcination (Xu et al., 2025).

The SEM (Supplementary Figure S2) and TEM images (Figure 1c) demonstrate that 3Co/CCN and CCN both possess a characteristic two-dimensional layered structure. To further investigate whether the Co species have been successfully incorporated into the framework of CCN, we performed an energy dispersive X-ray spectroscopy (EDS) element mapping analysis. As depicted in Figure 1d, it is evident that the 4 elements—Co, O, C and N—are uniformly distributed throughout the observation area, with no significant Co clusters detected. This suggests that the highly dispersed Co species have been successfully integrated into the covalent organic framework of CCN. The thickness of the samples was further examined using AFM. As illustrated in Figures 1e,f, the measured average thickness of 3Co/CCN is approximate 3.6 nm, which is 0.6 nm thinner than that of pristine CCN (4.2 nm). This nanoscale thickness, along with the Co-induced structural thinning, offers direct evidence of the successful fabrication of an ultrathin, two-dimensional sheet-like architecture of 3Co/CCN. The specific surface area is a crucial structural parameter that influences the catalytic performance of photocatalysts. As illustrated in Supplementary Figure S3, the CN-based materials all demonstrate IV-type N_2_ adsorption-desorption isotherms (Liu L. L. et al., 2025). The accompanying table in Supplementary Figure S3 presents the BET specific surface area data for 3Co/CCN, CCN, and CN. The specific surface area of 3Co/CCN is 58.9 m^2^·g^−1^, which exceeds that of CCN (35.2 m^2^·g^−1^) and CN (33.4 m^2^·g^−1^). This indicates that the incorporation of Co effectively enhances the specific surface area through by inhibiting the stacking of g-C_3_N_4_ layers during the thermal polymerization process, thereby facilitating greater exposure of active surface sites.

**FIGURE 2:**
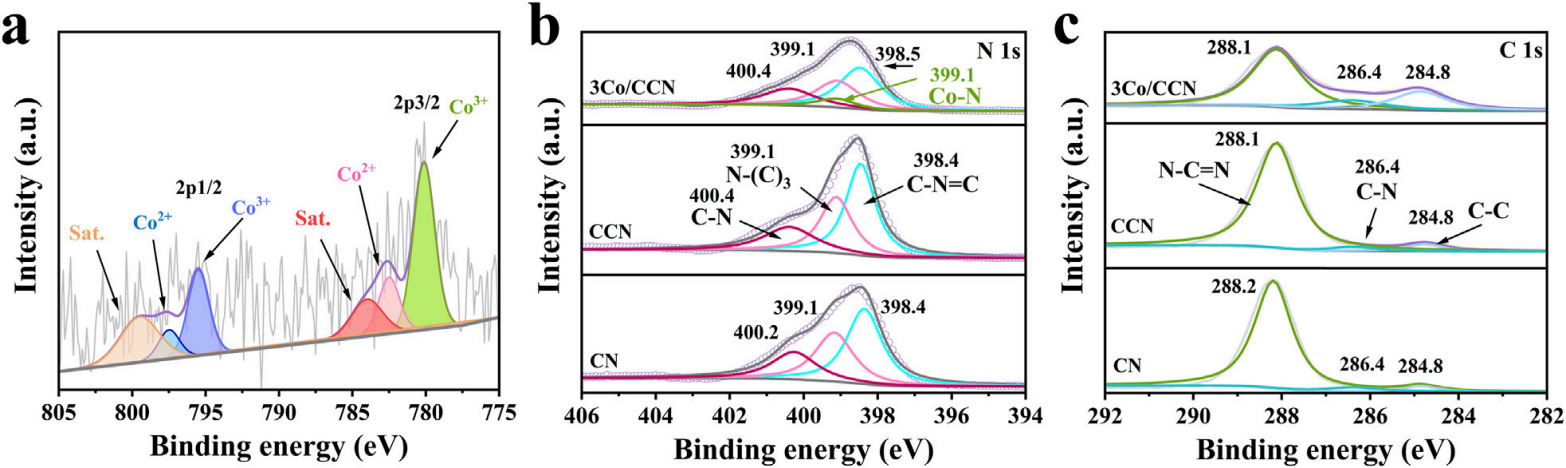
**(a)** High-resolution Co 2p spectrum of 3Co/CCN. **(b)** High-resolution N 1s and **(c)** C 1s spectra of 3Co/CCN, CCN and CN.

The local electronic structure and molecular coordination environment were further investigated using XPS. Figure 2a presents the high-resolution Co 2p XPS spectrum of the 3Co/CCN sample, which is utilized to ascertain the chemical oxidation state of the Co species. The peaks observed at 780.0 eV and 795.5 eV are attributed to Co^3+^ in the 2p3/2 and 2p1/2 states, respectively. Additionally, the peaks at 782.5 eV and 797.4 eV, along with the two satellite peaks at 784.0 eV and 779.5 eV, are associated with Co^2+^ in the 2p3/2 and 2p1/2 states. This analysis indicates the coexistence of both Co^3+^ and Co^2+^ within the catalyst (Tian et al., 2020). The lack of a peak indicative of the metallic state Co^0^ implies that Co is not present as nanoparticles. Instead, the Co^3+^/Co^2+^ centers are effectively integrated into the CCN photocatalytic material, functioning as the primary active sites for photocatalytic reactions. Furthermore, as illustrated in Figure 2b, the N 1s XPS spectra of CCN and CN both display three distinct peaks at 398.4 eV, 399.1 eV, and 400.4 eV, which correspond to sp^2^-hybridised nitrogen (C=N–C), tertiary nitrogen on the carbon (N–(C)_3_), and terminal amino groups (C–N–H), respectively. Notably, in the 3Co/CCN sample, the C–N=C peak shifted towards a higher binding energy of 398.5 eV, accompanied by a reduction in the intensity of the characteristic peaks when compared to the CCN. This observation suggests that the N atom within the CCN framework interacts with Co, leading to a decrease in the electron density of the N atom and a concomitant reduction in the intensity of the C–N=C peak (Jiang et al., 2024). This is further supported by the newly observed emergence of the Co–N peak at 399.1 eV in the 3Co/CCN N 1s XPS spectra (Zhang X. W. et al., 2026). However, the high-resolution C 1s spectra of 3Co/CCN, CCN, and CN samples (Figure 2c) consistently displayed three distinct components at binding energies of 288.2 eV, 286.4 eV, and 284.8 eV, corresponding to N=C–N, C–N, and C–C bonds, respectively (Zhang et al., 2023). The introduction of Co does not result in significant alteration in the XPS C 1s spectrum compared to the sample without Co, indicating that the incorporation of Co does not substantially affect the local coordination environment of the C atoms. Consequently, Co sites are likely to form coordination bonds with N atoms rather than C atoms, which is anticipated to enhance electron transfer between the CCN and Co centers.

**FIGURE 3:**
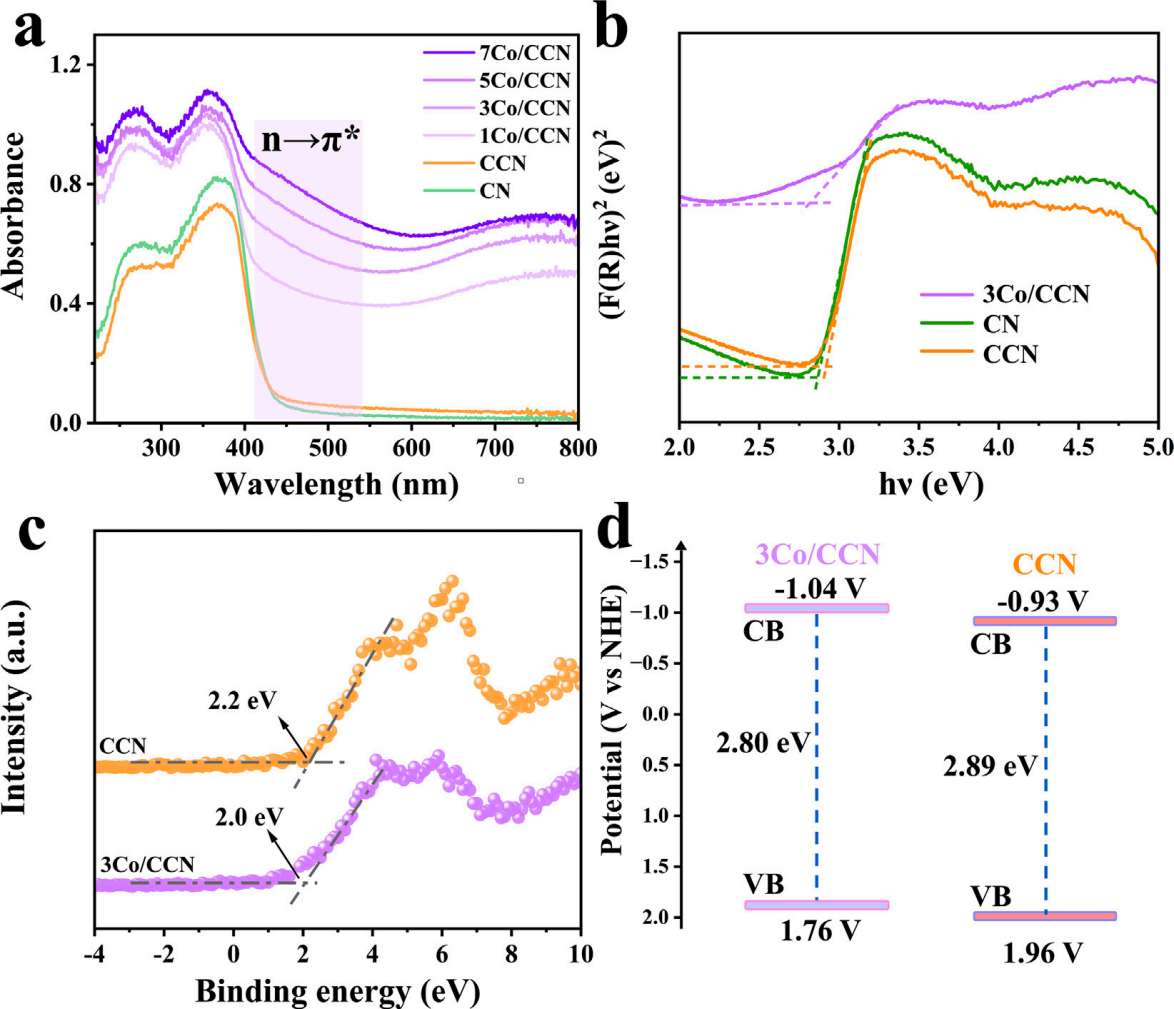
**(a)** UV-Vis absorption spectrum of Co/CCN with different Co loadings (1%, 3%, 5% and 7%), in addition to the spectra of CCN and CN without Co modification. **(b)** Kubelka-Munk plots utilized for band gap calculation of 3Co/CCN, CCN and CN. **(c)** Valence band XPS spectrum of 3Co/CCN and CCN. **(d)** Schematic band structure diagrams of 3Co/CCN and CCN.

The UV-Vis absorption spectrum and XPS valence band spectrum were employed to characterize the light response capabilities of the samples and determine their energy band structure. As illustrated in Figure 3a, the absorption threshold wavelengths for both CCN and CN samples were noted to be 450 nm, with the absorption edge remaining unchanged. Upon modification the samples with varying concentrations of Co, there was a notable enhancement in light absorption signals in both UV region and visible-light region, compared to the unmodified samples. The absorbance intensity exhibited a gradual increase in correlation with increasing Co content. This phenomenon is likely attributed to the introduction of Co–N_x_ species, which facilitates the n→π* transition of CCN, thereby extending the photocatalyst’s light absorption spectrum (Qin et al., 2026). The UV-Vis absorption spectrum data were further transformed using the Kubelka-Munk formula, resulting in the construction of Tauc plots, as illustrated in Figure 3b (Jiang et al., 2021). By extrapolating the linear segment of the curve to identify its intersection with the x-axis, the band gap values were determined. The calculations indicated that the band gap of CN was 2.86 eV, while that of CCN was determined to be 2.89 eV. Conversely, the band gap of 3Co/CCN was relatively narrower at 2.80 eV, indicating expanded visible-light range of 3Co/CCN. Additionally, the XPS valence band spectrum (Figure 3c) revealed that the valence band (VB) potential of CCN was 2.2 eV, while that of 3Co/CCN was 2.0 eV (Yu et al., 2017). Upon conversion them to the relative standard hydrogen electrode (RHE) potential, the VB potentials for CCN and 3Co/CCN were determined to be 1.96 V and 1.76 V, respectively. The detailed conversion process is provided in the Supplementary Material (Liang et al., 2021). This suggests that the incorporation of Co induces an upward shift in the VB’s top of the CCN. By integrating the VB potential values with the optical band gap data, the conduction band (CB) positions for both CCN and 3Co/CCN samples were determined to be −0.93 V and −1.04 V, respectively, as depicted in Figure 3d. The more negative CB potential for 3Co/CCN indicates an enhanced reduction capability of the photogenerated electrons in 3Co/CCN, thereby potentially enhancing the efficiency of photocatalytic degradation of DCF.

### 3.2 Charge separation

**FIGURE 4:**
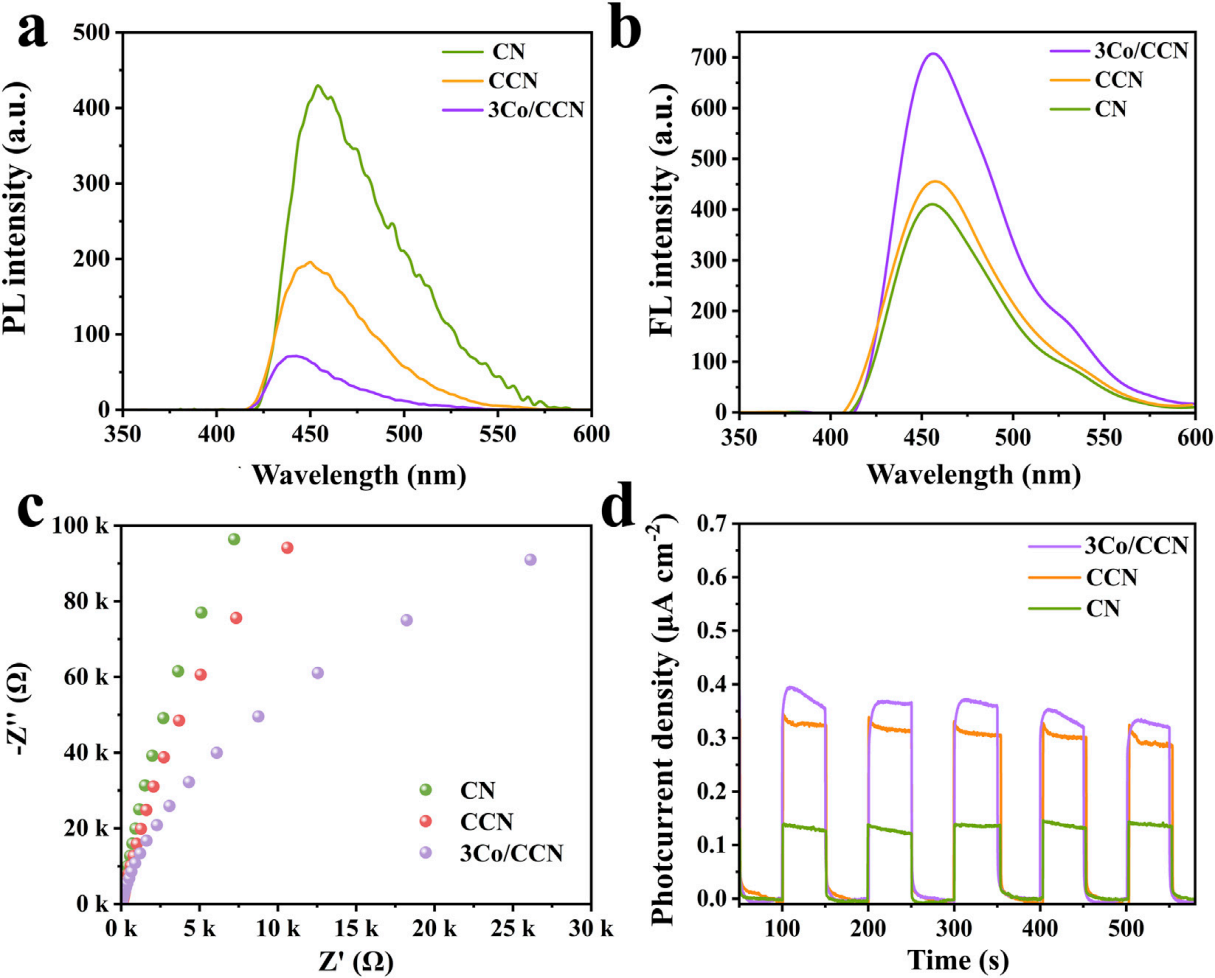
**(a)** Steady-state PL spectra of 3Co/CCN, CCN, and CN. **(b)** FL spectra associated with the generated hydroxyl radicals from 3Co/CCN and CCN. **(c)** EIS Nyquist plots for 3Co/CCN, CCN, and CN. **(d)** Transient photocurrent response curves of 3Co/CCN, CCN and CN under intermittent visible-light irradiation.

The efficiency of photogenerated charge separation in the photocatalyst is significantly positively correlated with its photocatalytic activity. The charge recombination behavior of the CN-based samples was characterized using PL spectra. When e^−^ transition from the lowest excited energy level back to the ground state and release energy in the form of photons, resulting in generating a PL signal. A higher PL signal implies a higher recombination rate of e^−^ and h^+^ (Wang et al., 2025), and *vice versa*. As shown in Figure 4a, the PL intensity of CN is the highest, followed by CCN. In contrast, the significantly lower PL intensity observed in 3Co/CCN indicates a suboptimal charge recombination that suggesting an enhanced charge separation. Additionally, the generation of hydroxyl radicals (·OH) plays a crucial role in the photocatalytic process since their formation reflects the effective participation of separated e^−^ and h^+^ in reactions with oxygen (O_2_) and water (H_2_O). Typically, the coumarin fluorescence method was employed to quantify the amount of ·OH radicals produced within the photocatalytic system (Tang et al., 2025). Figure 4b presents the FL spectra corresponding to the ·OH generated by different CN-based materials. The FL intensity of ·OH corresponding to 3Co/CCN is higher than that of CCN and CN, indicating that 3Co/CCN produced more ·OH under identical conditions. This observation indicates enhanced photocatalytic activity of 3Co/CCN. Further analysis of the charge separation properties of the samples was conducted through photoelectrochemical measurement. Figure 4c presents the EIS diagram, which was utilized to evaluate the charge transfer resistance of the semiconductor materials. The size of the semicircular diameter directly reflects the numerical value of the charge transfer resistance. It is evident that the semicircular diameter of the 3Co/CCN sample is the smallest, indicating it has a lower interface resistance (Chen et al., 2024). This may be attributed to the introduction of Co active sites, which enhance the efficient separation of photogenerated charge carriers and consequently facilitate the transfer of photogenerated electrons. Figure 4d presents the transient photocurrent curves for 3Co/CCN, CCN, and CN. When the visible-light irradiation was initiated, the photocurrent density raised rapidly and reached a steady-state plateau. When the light source was turned off, the photocurrent density quickly returned to the baseline, without exhibiting an obvious photocurrent hysteresis phenomenon (He et al., 2026). The photocurrent density of 3Co/CCN surpassed that of both CCN and CN, and the photocurrent response remained stable without significant attenuation after five cycles of on-off light irradiation. This indicates an excellent charge separation capability and photochemical stability following the incorporation of Co.

### 3.3 Photocatalytic activities

**FIGURE 5:**
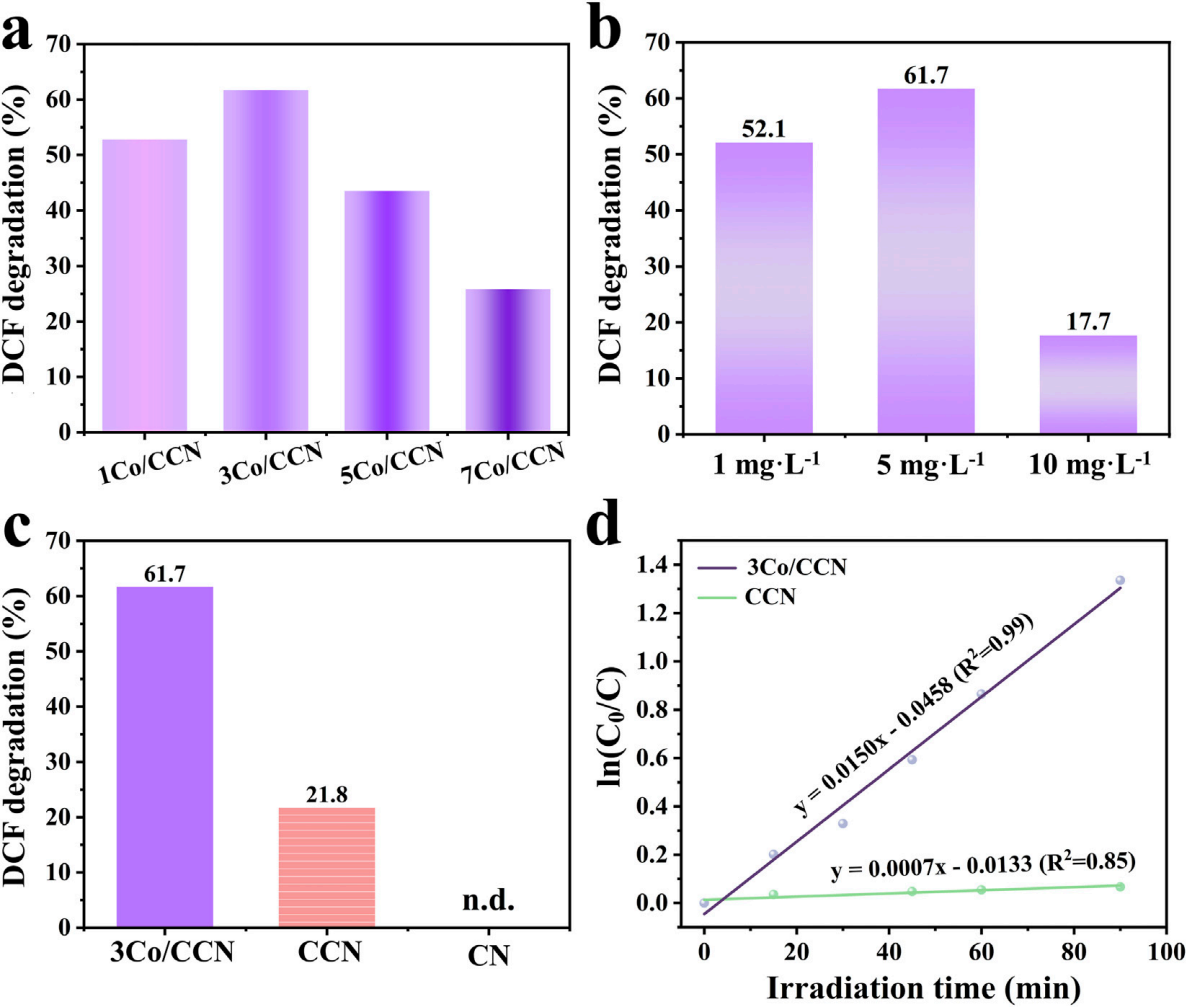
**(a)** DCF degradation using Co/CCN with different Co loading (1%, 3%, 5% and 7%). **(b)** The influence of initial DCF concentrations (1, 5, and 10 mg·L^-1^) in 3Co/CCN degradation system. **(c)** Comparison of DCF degradation over 3Co/CCN, CCN and CN (n.d. = no detectable degradation). **(d)** Pseudo-first-order kinetic plots for DCF degradation over 3Co/CCN and CCN.

As depicted in Figure 5a, the degradation of DCF within 1 h initially increased and then decreased as the mass percentage of added Co was incrementally increased from 1% to 7%. The sample with 3% Co (3Co/CCN) exhibited the highest degradation, suggesting that the incorporation of an optimal 3% Co could enhance the photocatalytic activity of CCN for DCF degradation, which was subsequently utilized in further experiments. In contrast, as depicted in Supplementary Figure S4, DCF itself did not exhibit significant photodegradation after 1 h of visible-light irradiation, suggesting that the observed degradation was solely attributable to the photocatalytic activity of the catalyst, rather than self-photodegradation of DCF. Additionally, the data presented in Figure 5b reveal that at a DCF concentration of 5 mg·L^−1^, 3Co/CCN exhibited optimal photocatalytic performance. However, this degradation markedly decreased to 17.7% when the DCF concentration was increased to 10 mg·L^-1^. This finding suggests that 3Co/CCN achieves its optimal performance primarily in low-concentration DCF solutions, likely due to the saturation of active sites on the 3Co/CCN surface at higher DCF concentrations. What’s more, a comprehensive comparative analysis of the adsorption and degradation performance of various CN-based materials was conducted. As illustrated in Supplementary Figure S4, following equilibrium in the dark, the adsorption of DCF on 3Co/CCN was 18.4%, significantly surpassing the adsorption on CCN (10.6%) and CN (10.1%). This enhanced adsorption performance is likely attributable to the modified Co sites, which may promote selective adsorption of DCF through Co–Cl interactions (Min et al., 2021; Ren et al., 2024). Furthermore, as depicted in Figure 5c, under visible-light irradiation, 3Co/CCN achieved a degradation rate of 61.7% for DCF, approximately 2.8 times higher than the 21.8% degradation observed with CCN, while CN did not exhibit any visible-light degradation of DCF. The degradation process was examined using pseudo-first-order kinetic plots, revealing a linear relationship as shown in Figure 5d, described by the equation (Huang et al., 2024):

$$\ln\left(C_{0}/C\right)=kt$$

The kinetic rate constant (*k*) for 3Co/CCN was determined to be 0.015 min^−1^, which was 21.4 times greater than that of CCN at 0.0007 min^−1^ (Supplementary Figure S6). This indicates that the incorporation of Co active sites significantly enhances the adsorption and photocatalytic degradation capability for DCF.

**FIGURE 6:**
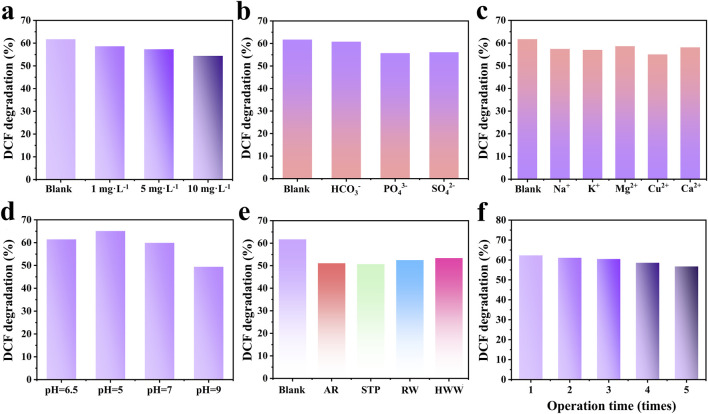
The photocatalytic activity of 3Co/CCN for DCF degradation under various influencing factors: **(a)** concentration of HA after filtration, **(b)** inorganic cations, **(c)** inorganic anions, **(d)** pH values, and **(e)** different actual water matrices and wastewater samples. **(f)** Cycling stability test of 3Co/CCN for DCF degradation over 5 cycles of testing.

Figures 6a–c presents a comparative analysis of the influence of environmental interference factors on the degradation of 5 mg·L^−1^ DCF. The results indicated that the presence of humic acid, inorganic cations, and anions in the system had a relatively minor impact on the degradation of DCF. However, variations in pH significantly affected the photocatalytic degradation of DCF (Figure 6d). The initial pH of the 5 mg·L^−1^ DCF solution was measured at 6.5. Degradation was found to be enhanced under acidic conditions (pH 5) and inhibited under alkaline conditions (pH 9). This behavior may be attributed to the presence of different ionic forms of DCF at varying pH levels. Given that the pKa of DCF is 4.15, when the pH exceeds 4.5, DCF predominantly exists in its ionic form, and as the pH increases, the anionic form (DCF^−^) becomes more prevalent (Martínez-Rodriguez et al., 2025; Wang et al., 2024). Furthermore, the surface charge of 3Co/CCN is affected by changes in pH, as evidenced by Zeta potential measurements shown in Supplementary Figure S7. At pH values above 8, the surface of 3Co/CCN becomes negatively charged. The negative charges of both DCF and 3Co/CCN at pH 9 result in electrostatic repulsion, which may inhibit DCF’s adsorption onto the 3Co/CCN surface, thereby impacting the photocatalytic degradation process. Consequently, when treating alkaline wastewater containing DCF, it is recommended to adjust the pH to neutral or slightly acidic conditions prior to photocatalytic degradation. The potential application of 3Co/CCN in complex aquatic environments was further evaluated by collecting samples from various sources, including wastewater treatment plants (STP), river water (RW), agricultural runoff (AR), and hospital wastewater (HWW). These samples were supplemented with a 5 mg·L^−1^ DCF standard solution to simulate real wastewater conditions containing DCF. As depicted in Figure 6e, the degradation of DCF within the 3Co/CCN system across these complex matrices did not exhibit a significant reduction compared to the deionized water system, indicating a robust resistance to interference and suggesting considerable application potential. Furthermore, a cyclic stability test was conducted on 3Co/CCN (Figure 6f; Supplementary Figure S8), demonstrating that the material maintained substantial photocatalytic activity even after five testing cycles, with no discernible alterations in its structure.

## 4 Conclusion

In summary, we developed a simple and effective approach for the dispersion of CoPc through hydrogen bonding of CA, leading to the formation of a Co-CA-urea supermolecule precursor. This precursor is subsequently via a one-step thermosetting polymerization into a highly dispersed Co-modified CCN photocatalyst. This 3Co/CCN catalytic material demonstrated remarkable improvements in the adsorption performance and photocatalytic activity of DCF, attributed to structural regulation and Co^2+^/Co^3+^ active site design. The degradation kinetics were enhanced 21.4 times compared to pure CCN. Extensive characterization analyses revealed that the highly dispersed Co sites not only extended the range of visible-light absorption but also acted as centers for charge separation, thereby enhancing the separation and migration of photogenerated carriers. This study elucidates the crucial role of highly dispersed metal sites in the degradation of chlorinated organic pollutants, and provides an important theoretical foundation and technical support for the development of high-performance visible-light responsive photocatalysts for water remediation.

## Data Availability

The raw data supporting the conclusions of this article will be made available by the authors, without undue reservation.
