# Supplementary material for: Cobalt-modified graphitic carbon nitride nanosheets for the photocatalytic degradation of diclofenac
Source: Front Chem. 2026 Jul 27;14:1901405. doi: 10.3389/fchem.2026.1901405 (PMC13454458; doi:10.3389/fchem.2026.1901405)
Supplement: Supplementary file 1 [file DataSheet1.docx]

Supplementary Material

# 1 SUPPLEMENTARY EXPERIMENTAL DETAILS

**1.1. Photocatalytic activity**

The photocatalytic degradation activity of the catalyst was evaluated via the degradation of DCF in a 100 mL glass beaker. In a typical experiment, 50 mg of the catalyst was added to 50 mL of a DCF solution with a concentration of 5 mg·L^-1^. The mixture was stirred in the dark for 30 min to achieve adsorption-desorption equilibrium. Subsequently, a 150 W white LED light source (with a light intensity of 21.6 mW·cm^-2^) was activated, and stirring was maintained to initiate the photocatalytic reaction. At the predetermined time intervals, 5 mL of the reaction mixture was sampled and immediately filtered through a 0.22 μm aqueous microporous membrane. The concentration of DCF in the filtrate was quantified using an UV-Vis spectrophotometer.

**1.2. Interference tests under diverse scenarios**

Photocatalytic degradation experiments of DCF with varying initial concentrations were conducted, where substrate concentration served as the variable, while all other operations and reaction parameters were consistent with the standard experimental protocol.

Anion-cation interference experiments were designed based on the concentration levels of common anions and cations in natural water bodies. All operational steps and parameters were identical to those of the benchmark photocatalytic degradation experiment, except that the corresponding anions and cations were individually added to each parallel system following the achievement of adsorption-desorption equilibrium.

Photocatalytic degradation experiments of DCF with varying concentrations of humic acid were performed as follows: Humic acid at gradient concentrations was pre-added to the 5 mg·L^-1^ DCF solution, followed by stirring in the dark for 30 min to establish a complete complexation–dissolution equilibrium between humic acid and DCF. Subsequently, the suspension was filtered through a 0.22 μm aqueous-phase microporous membrane to remove insoluble solids. All subsequent procedures were conducted in strict accordance with the protocol established for the benchmark photocatalytic degradation experiment.

Photocatalytic degradation experiments of DCF were conducted under varying initial pH conditions: the 5 mg·L^-1^ DCF solution was pre-adjusted to the target pH values using appropriate acid/base solutions, and all other experimental procedures and reaction parameters were maintained strictly in accordance with those specified in the reference photocatalytic degradation experiment.

Verification experiments were conducted using real natural water matrices: 5 mg·L^-1^ DCF solutions were prepared by spiking DCF into four distinct natural water samples collected from representative environmental settings. All experimental procedures and reaction parameters were maintained strictly in accordance with those specified in the reference photocatalytic degradation experiment, with the sole modification being the substitution of ultrapure water with the respective natural water matrix as the reaction medium.

# 2 SUPPLEMENTARY CHARACTERIZATIONS

**2.1. Electrochemical reduction measurement**

The electrochemical test was conducted in a traditional three-electrode system. The prepared film electrode served as the working electrode, a platinum sheet (99.9%) as the counter electrode, Ag/AgCl as the reference electrode, and the electrolyte was a 0.2 M Na_2_SO_4_ solution. The electrochemical performance of a series of photocatalysts was tested using the IVIUM V13806 electrochemical workstation (Ivium Technologies B.V., V13806, NLD). All experiments were carried out at room temperature (approximately 25 °C).

**2.2. XPS valence band spectrum data processing**

The XPS valence band position (E_VB-XPS_) was converted to the E_VB_ relative to the normal hydrogen electrode (NHE) using the following equation.

$$E_{\mathrm{VB}}=\Phi+E_{VB-XPS}-4.44$$

where E_VB_, Φ and E_VB-XPS_ represents valence band position vs. NHE, the electron work function of the analyzer and the value of valence band maximum.

# 3 SUPPLEMENTARY FIGURE S1

**Supplementary Figure 1.** UV-Vis absorption spectrum of CoPc, CA+CoPc, and CA.

# 4 SUPPLEMENTARY FIGURE S2

**Supplementary Figure 2.** SEM images of (a) 3Co/CCN and (b) CCN.

# 5 SUPPLEMENTARY FIGURE S3

**Supplementary Figure 3.** Nitrogen adsorption-desorption isotherms of 3Co/CCN, CCN and CN (inset: BET specific surface area).

# 6 SUPPLEMENTARY FIGURE S4

**Supplementary Figure 4.** The UV-Vis absorption spectrum of DCF before (DCF) and after 1 h of self-photolysis (DCF_1 h_).

# 7 SUPPLEMENTARY FIGURE S5

**Supplementary Figure 5.** Comparison analysis of 3Co/CCN, CCN, and CN for DCF adsorption.

# 8 SUPPLEMENTARY FIGURE S6

**Supplementary Figure 6.** Comparison of kinetic rate constant *k* values between 3Co/CCN and CCN.

# 9 SUPPLEMENTARY FIGURE S7

**Supplementary Figure 7.** Zeta potential distribution profile of 3Co/CCN at varying pH levels.

# 10 SUPPLEMENTARY FIGURE S8

**Supplementary Figure 8.** XRD patterns of 3Co/CCN before and after photocatalytic reaction.
